# JN. 1 and cardiac-related clinical manifestations: a current public health concern

**DOI:** 10.3389/fcvm.2024.1488226

**Published:** 2024-12-09

**Authors:** Sangeeta Chhotaray, Pralaya Kumar Sahoo, Suman Kumar Mekap, Soumya Jal, Gurudutta Pattnaik

**Affiliations:** ^1^ School of Paramedics and Allied Health Sciences, Centurion University of Technology and Management, Bhubaneswar, India; ^2^ School of Pharmacy and Life Sciences, Centurion University of Technology and Management, Bhubaneswar, India

**Keywords:** JN-1 virus, RNA virus, cardiovascular conditions, public health, COVID-19

## 1 Introduction

The current COVID-19 pandemic is a worldwide emergency because of its rapid spread and high mortality rate, resulting in considerable disruptions. The virus responsible for COVID-19, brought the world to a halt, presenting 2020 with the coronavirus pandemic is still on the rise around the world (1). Individuals afflicted with COVID-19 have the potential to get pneumonia, Severe manifestations of acute respiratory distress syndrome (ARDS) and the collapse of numerous organs (2–5). High fatality rate due to pandemic had a worldwide impact on the lives of people, resulting in substantial stresses on daily life (6). Despite extensive reports on mental health during the COVID-19 pandemic, there is a lack of research on the impact on individuals with moderate cardiac and psychological issues. The World Health Organisation (2020) states that individuals with chronic conditions and those who fail to follow COVID-19 protection protocols are at a higher risk of infection. The COVID-19 pandemic, characterized by its unpredictability and the implementation of lockdowns and physical distancing, may heighten the risk of mental health issues and exacerbate existing health problems (7). The relentless evolution of this virus variants remains a formidable challenge to global public health, thus prompting significant concern among health authorities due to emergent strains (8).

Recently the World Health Organisation has included a new strain of COVID-19, JN.1, in its database of “Variant Of Interest”, which was initially identified in September, 2023 in 12 countries, with the largest occurrences observed in Canada, France, Singapore, Sweden, the UK, and the US (9). This “Variant Of Interest” (VOI) was recently identified as distinct sub-lineage stemming from the BA.2.86 variant. Noteworthy mutations include R3821K in ORF1a, L455S in the spike protein, and F19l in ORF7b, to characterize JN.1, the prevalence of which is steadily surging worldwide, signalling a remarkable competitive advantage. While differing from its parent variant, BA.2.86, in terms of infectivity and immune evasion, current evidence does not support heightened pathogenicity associated with it however, the augmented immune evasion capabilities raise concerns about potential waves of infections, particularly among individuals previously exposed to earlier variants (10). And JN.1 also has a distinct genetic variation that belongs to the BA.2.86 lineage and is characterised by particular genetic alterations. Based on data obtained from the website cov-spectrum (https://cov-spectrum.org), the global detection of the JN.1 variant (including the JN.1 variant and all its descendant variants) has reached a total of 16,604 sequences as of 26 December 2023. This accounts for the largest proportion (47.9%) and demonstrates a consistent upward trend (11).

So, the main aim of this research is to examine the prevalence of the JN. 1 infection in the development and progression of cardiac-related clinical disorders, including its possible influence on the prevalence of heart disease. The aim seeks to elucidate the molecular and physiological pathways connecting JN. 1 to cardiovascular health, so improving comprehension of its role in public health concerns, facilitating early diagnosis, and guiding treatment approaches to reduce the risks associated with heart problems.

The emergence of a new variant may significantly impact individual health and increase stress reminiscent of the previous pandemic. Research shows that arterial hypertension is linked to a greater susceptibility to SARS-CoV-2 infection, worsened disease severity, and higher mortality rates from COVID-19 (12, 13). Moreover, experimental research indicated that critical pathophysiological pathways of hypertension, such as the stimulation of the renin-angiotensin system (RAS), may contribute to COVID-19 (14, 15). And the prior analysis overlooked the link between pandemic-induced hypertension and cardiac problems, leading to a high number of deaths during the pandemic. Vascular dysfunction is a key contributor to various diseases, including hypertension, diabetes, and obesity, which significantly increase the risk of COVID-19-related mortality.

## 2 COVID-19 and cardiac relation

A comprehensive literature analysis was conducted to locate scholarly articles on COVID-19, which is caused by the severe acute respiratory syndrome coronavirus 2 (SARS-CoV-2). The search was performed using the databases of the World Health Organisation (WHO) and the American Heart Association, covering the period from March 2020 to August 2022. A prominent academic health system in New York City, using a retrospective observational design reported 45,398 individuals to be diagnosed with COVID-19 between March 2020 and August 2022. During the 6-month follow-up period, it was shown that 20.6% of patients who were hospitalised with COVID-19 had new and ongoing high blood pressure (16). The history of hypertension was characterised by individuals who fulfilled at least one of the three criteria before to the COVID-19 pandemic: (1) Patients had an average blood pressure of above 140 mmHg systolic or 90 mmHg diastolic blood pressure two weeks before being diagnosed with COVID-19. Please refer to the sensitivity analysis using cutoffs of 130/80 mmHg. (2) Patients had a previous diagnosis of hypertension based on the ICD-10 code before being diagnosed with COVID-19. (3) Patients were prescribed at least one antihypertensive medication at the time of COVID-19 diagnosis.

## 3 Discussion

The genetic material of the COVID-19 virus has four crucial structural proteins: spike (S), envelope (E), matrix/membrane (M), and nucleocapsid (N), in addition to a group of supplementary proteins (17, 18). Among them spike proteins play a crucial role in facilitating the entry of coronaviruses into cells and causing infection. The functional receptor for COVID-19 has been identified as angiotensin-converting enzyme 2 (ACE2) (19). The primary physiological purpose of ACE2 is linked to its metalloprotease activity, which is crucial in controlling and processing Renin-Angiotensin system (RAS) circulating peptides. ACE2 acts as a counter regulatory mechanism to counteract the effects of angiotensin II (Ang II) produced by ACE (20). In order to infect hosts, the virus uses its receptor, ACE2. As RAS has a series of receptors, enzymes, and peptides that play an essential role in maintaining fluid and electrolyte balance as well as regulating blood pressure through its two pathways: the pressor pathway and the depressor pathway (21). In the last two decades, ACE2 and its heptapeptide product angiotensin Ang 1–7 have been increasingly acknowledged as counterregulatory modulators of the classical RAS through the activation of the Mas receptor (MasR) (22). ACE2 primarily transforms Ang II into Ang 1–7, subsequently activating the MasR signalling pathway, which exerts protective downstream effects on the microcirculatory environment. And Ang II functions through the Ang II type-1 receptor (AT1R) to elicit vasoconstriction, stimulate inflammatory cytokine production, and promote extracellular matrix creation. Ang II further promotes adrenal aldosterone synthesis, resulting in salt and fluid retention and an elevation in blood pressure. Conversely, Ang 1–7, through the MasR, promotes vasodilation and suppresses the synthesis of proinflammatory cytokines, counteracting the effects of Ang II (23).

**Figure 1:**
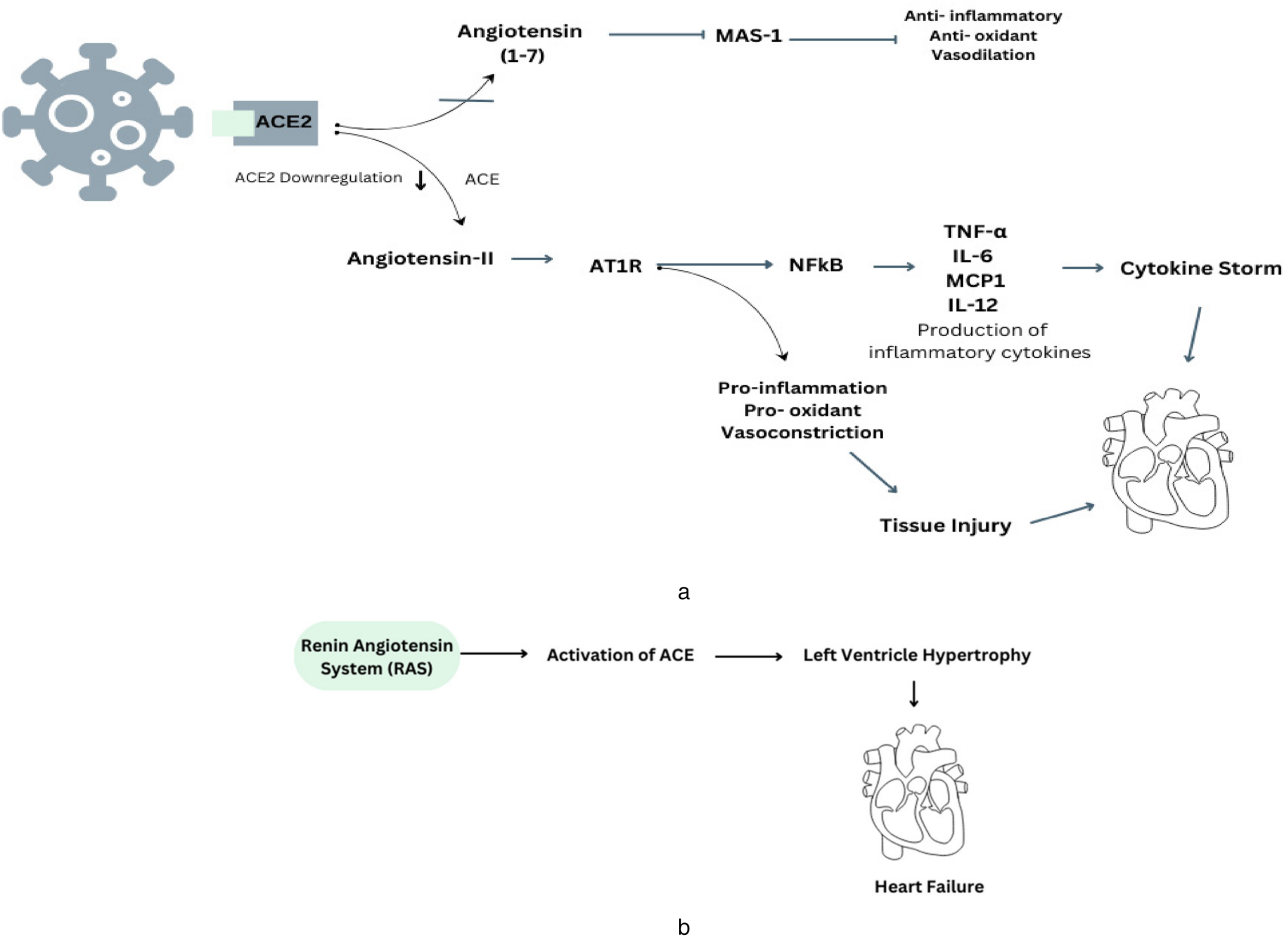
**(a)** This diagram illustrates the underlying mechanisms involved in the development of COVID-19 and how they connect with the renin-angiotensin system, specifically in relation to the regulation of ACE2. **(b)** The presence of RAS-mediated ACE production contributes to the development of cardiovascular problems in cases of hypertension.

Therefore, the viral invasion through ACE2 leads to a decrease in the presence of ACE2 on the cell membrane and a simultaneous reduction in the enzymatic function of ACE2 in the RAS. Thus, the virus may cause a decline in Ang (1–7) levels, so tilting the equilibrium towards the vasoconstrictor aspect of the RAS, potentially resulting in the loss of cardiovascular stability in individuals with COVID-19 (24). Another mechanism that can lead to cardiovascular complications is the cytokine storm (Figure 1a). Cytokine storm syndrome (CSS) is linked to advanced and severe cases of COVID-19, and its pathophysiological causes can be attributed to several pathways. This infection associated to a hyperinflammatory response, characterized by the release of excessive cytokines by the immune system. This response can lead to complications like acute respiratory distress syndrome (ARDS), organ failure, and increased mortality (25). The primary receptor for entry into human cells is ACE2, a transmembrane glycoprotein part of the renin-angiotensin-aldosterone system (RAAS). The ACE2 receptor, which cleaves angiotensin I and II, produces peptides with RAAS-antagonistic properties. SARS-CoV-2 binds to the ACE2 receptor, leading to its internalization or cleavage by cellular proteases, resulting in tissue downregulation of ACE2. The loss of ACE2-mediated anti-inflammatory, antithrombotic, and anti-fibrotic effects, along with the upregulation of the angiotensin II-AT1 axis, may contribute to the development of the cytokine storm and thrombo-inflammatory state associated with COVID-19 (26). And the initial mechanism also involves down-regulation of ACE2, resulting in an elevated level of Ang II due to the lack of conversion into Ang-(1–7). Therefore, the unregulated activation of the ACE/Ang II/AT1R pathway leads to elevated levels of pro-inflammatory cytokines, such as IL-1, IL-6, and TNF-α, which are further enhanced by the stimulation of both innate and adaptive immunological responses (27). The virus can also augment the DNA-binding capability of nuclear factors, such as NF-KB, potentially leading to an increase in mRNA transcription of various interleukins.

Therefore, existing evidence indicates ACE2 is a key factor in cardiovascular disease, especially heart failure. It is present in various cells, including epithelial cells, cardiac myocytes, vascular smooth muscle, endothelial cells, and brain tissues (28). Prolonged ACE2 inhibition can increase cardiac Ang II levels, left ventricular wall thicknesses, interstitial collagen fraction area, and cardiomyocyte hypertrophy (Figure 1b). This suggests that ACE2 is crucial in the development of cardiovascular disease (29).

## 4 Limitations

### 4.1 Confounding variables

Confounding factors, like lifestyle, genetic predisposition, or pre-existing conditions, may not have been sufficiently controlled, thereby affecting the outcomes.

### 4.2 Geographical and ethnic limitations

If the study is localized to a certain place, the results may not be applicable to other populations with varying environmental or genetic characteristics.

## 5 Future directions

### 5.1 Global collaborative studies

Conducting studies in multiple countries or regions would help determine if the cardiac effects of JN. 1 are consistent across different populations, or if regional factors (like diet, healthcare access) play a role.

### 5.2 Public health strategies

The study's results should inform public health programs focused on monitoring and managing the cardiac-related clinical symptoms of JN. 1. This may entail enhanced screening for cardiac conditions in communities identified as being impacted by JN. 1.

## 6 Conclusion

COVID-19 has become a significant respiratory infection and a major cause of cardiac-related symptoms, posing a complex public health dilemma. Understanding the processes behind cardiac problems is crucial for developing effective therapies and preventative methods. The surge in COVID-19 cases and worldwide infections is likely unavoidable. It is advised to stay alert for JN.1 lineages of Omicron, as cardiovascular problems may arise from viral invasion, inflammatory reactions, and drug use. Despite COVID-19 vaccinations, cardiovascular problems remain a concern. It is essential to closely monitor the epidemiology of newly identified variations and lineages to detect any rise in severe illness outcomes. Assessing vaccine and antibody-based therapies against these variants is crucial, as well as developing next-generation vaccines, improved vaccines, and new monoclonal antibodies to prevent the spread of evolving SARS-CoV-2 variants and lineages.
